# Re-assessing the Psychometric Properties of Stress Appraisal Measure in Ghana Using Multidimensional Graded Response Model

**DOI:** 10.3389/fpsyg.2022.856217

**Published:** 2022-05-19

**Authors:** Medina Srem-Sai, Frank Quansah, John Elvis Hagan, Francis Ankomah, James Boadu Frimpong, Prosper Narteh Ogum, Thomas Schack

**Affiliations:** ^1^Department of Health, Physical Education, Recreation and Sports, University of Education, Winneba, Ghana; ^2^Department of Educational Foundations, University of Education, Winneba, Ghana; ^3^Department of Health, Physical Education and Recreation, University of Cape Coast, Cape Coast, Ghana; ^4^Neurocognition and Action-Biomechanics-Research Group, Faculty of Psychology and Sports Science, Bielefeld University, Bielefeld, Germany; ^5^Department of Education and Psychology, University of Cape Coast, Cape Coast, Ghana; ^6^Department of Education, Seventh Day Adventist (SDA) College of Education, Asokore, Ghana

**Keywords:** football players, Ghana Premier League, graded response model, stress, stress appraisal, validation

## Abstract

Despite the widespread use of the stress appraisal measure questionnaire in sport psychology literature, information on the psychometric properties of this survey instrument across different cultures and samples is still lacking. This study sought to validate the stress appraisal measure among male football players in the Ghana’s Premier League using the multidimensional item response theory. The descriptive cross-sectional survey design was adopted to recruit 424 footballers from the 2020/2021 Ghana Premier League season using the census approach. The 28-item Stress Appraisal Measure was used to assess six (6) appraisal mechanisms under primary and secondary cognitive appraisals. The ordered polytomous item response theory was used for analyzing the data. The study found that although some items were problematic, the majority of them were found to have good item parameters, effective scale option functioning, and provided adequate empirical information in the measurement of stress appraisal. This research concluded that the stress appraisal measure has promising applicability among male footballers who participated in the premier league in Ghana. Future researchers are encouraged to re-validate the stress appraisal measure with a different sample to contribute to the understanding of the applicability of the instrument in non-western populations.

## Introduction

The Stress Appraisal Measure (SAM) emerged as one instrument that gained prominence in assessing the cognitive appraisal of stress across different samples (Peacock and Wong, 1990; Rowley et al., 2005; Durak and Senol-Durak, 2013), despite the existence of several other scales such as the Stress Appraisal Inventory for Life Situations (Groomes and Leahy, 2002), the Primary Appraisal Checklist (Dewe, 1993), Primary Appraisal Secondary Appraisal Scale (Gaab et al., 2003), Daily Stress Inventory (Brantley et al., 1987), the Perceived Stress Scale (Cohen et al., 1983), and the Lifestyle Appraisal Questionnaire (LAQ; Craig et al., 1996). The SAM is a multidimensional instrument that measures both primary and secondary cognitive appraisals as specified by the transactional model of stress and coping propounded by Lazarus and Folkman (1984). The primary appraisal which involves the evaluation of anticipatory harm or benefit resulting from interacting with an individual or environment (Folkman et al., 1986) consists of *threat, challenge* and *centrality* (Peacock and Wong, 1990). Conversely, the secondary appraisal which involves the evaluation of all the actions that can be done to mitigate the negative effects of the anticipatory harm or enhance the chances of benefit (Folkman et al., 1986) consists of *controllable by self, controllable by others*, and *uncontrollable by anyone* (Peacock and Wong, 1990).

Even though the SAM has been in existence for over three decades, few studies have assessed its validity across different geographical contexts (Peacock and Wong, 1990; Rowley et al., 2005; Durak and Senol-Durak, 2013). For instance, Peacock and Wong (1990) assessed the construct validity of the SAM with factor analyses using 151 undergraduate students. Peacock and Wong (1990) found that the psychometric properties of the SAM appeared to be good for the study sample and measured six independent dimensions. This notwithstanding, Peacock and Wong (1990) stated that “there is the need for further psychometric data, especially those obtained in differing contexts and with a broader range of respondents” (p. 235). Another study by Rowley et al. (2005) employed an exploratory factor analysis (EFA) and confirmatory factor analysis (CFA) to validate the SAM using 172 adolescents. Rowley et al. (2005) observed that the three-factor model for the SAM for adolescents fit extraordinarily well for *threat*, *challenge* and *resources* but not *centrality*. Durak and Senol-Durak (2013) also assessed the psychometric properties of the SAM with three different unrelated samples (i.e., two distinct groups involving university students and adults), with the data subjected to parallel and principal axis factor analyses as well as convergent and discriminant validity tests. Durak and Senol-Durak findings showed that the psychometric properties of the SAM was satisfactorily appropriate when utilized in Turkish samples. However, the authors recommended that future studies should consider other samples who experience different forms of stressors and in different cultural settings to ascertain its generalization and applicability.

Despite the documented pervasiveness of stressful experiences (e.g., high intensive matches, frequent traveling, unfamiliar sleeping environments, and short recovery phase) among professional football players (Dupont et al., 2010; Kristiansen et al., 2012; Nédélec et al., 2012), it is surprising that no study has assessed the validity of the SAM using this sample. This creates a big vacuum in the literature that needs urgent attention, especially when the SAM has been utilized by several scholars within sport psychology research (see Gan and Anshel, 2006; Gan et al., 2009; Nicholls et al., 2016; Nicholls and Perry, 2016). Moreover, findings from previous validation studies (Peacock and Wong, 1990; Rowley et al., 2005) may yield varying applicability in other contexts such as Africa given the collectivist nature of its setting as opposed to the individualistic nature of Canada where Peacock and Wong’s (1990) study was conducted, and probably Turkey (Durak and Senol-Durak, 2013) where the religious beliefs and practices may also vary. Therefore, how a sample drawn from an African context like Ghana would understand or interpret the items of the SAM may differ from those from other jurisdictions. Hence, the applicability of the SAM in other geographical boundaries like Ghana may not be well understood. To date, the psychometric properties of the SAM have not been tested in many non-Western countries except Turkey, with none documented in Africa. Adapting the SAM in the African context might provide useful information on stress appraisal situations for appropriate coping interventions, especially in professional football where stressful experiences among players are common. Besides, inconsistencies in the findings from previous validation studies (Peacock and Wong, 1990; Rowley et al., 2005; Durak and Senol-Durak, 2013) suggest that further studies are warranted to ascertain the applicability of the SAM using different samples with a modern non-sample dependent measurement procedure like the item response theory (IRT).

The multidimensional graded response model, which is one of the forms of IRT models, is a powerful approach to modeling used to assess the properties of survey instruments with ordered responses (Raykov and Marcoulides, 2011). This approach to validation would provide robust and objective information on the psychometric properties of SAM relative to those provided by previous studies. More specifically, previous studies adopted a factor analytic approach (classical test theory, CTT) which largely focuses on inter-item covariances as well as the linear relation between item response and factor scores. Item response theory (IRT) models, in contrast, evaluate the non-linear relation between item responses and latent traits (Rasch, 1993). The use of the IRT models, especially the multidimensional graded response model, offers essential information (e.g., response category functioning) that the CTT approaches do not provide. Several other scholars have recommended the use of the IRT models for the validation of survey instruments (Samejima, 1997; Embretson and Reise, 2000; Kamata and Bauer, 2008).

**FIGURE 1:**
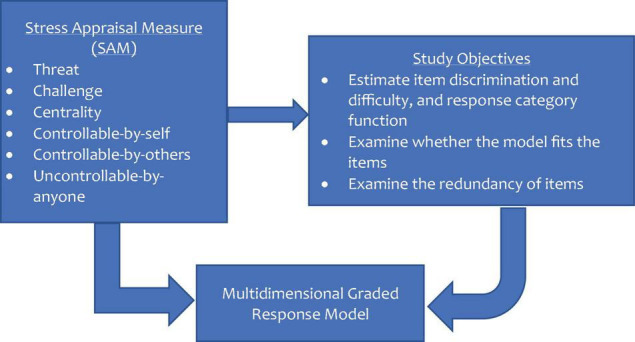
Study flowchart.

Therefore, the intent of this research was to validate the SAM among male football players in the Ghana Premier League (GPL) using the multidimensional item response theory. Particularly, the study assessed the quality of the items by identifying the trends in the responses. Taken together, this research examined the following: (1) the item parameters (discrimination and difficulty indices of the items) to find out whether the items are able to discriminate between participants with a high level of the construct and those with low level, as well as to understand how the 5-point response category (*not at all*, *slightly*, *moderately*, *considerably*, and *extremely*) function; (2) the item level fit to evaluate whether the model fit the items on the SAM and the extent to which the items play a significant part of the measure; and (3) the item information function to ascertain whether there are redundant items present on the instrument. A study flowchart was also designed to provide a visual understanding (see Figure 1).

## Materials and Methods

### Participants’ Characteristics

Originally, a sample projection of 500 was made because (1) scholars in the measurement field have suggested that using 500 cases in multidimensional item response theory analysis provide accurate estimation of parameters (see Forero et al., 2009; Jiang et al., 2016) and (2) anecdotal information from the various clubs during familiarization visit by the investigators showed that the players were a little over 500 and thus, it was necessary to target all the players (i.e., through census approach) to ensure accurate estimations. However, one of the football clubs and few players in some of the teams opted out of the study resulting in a final sample of 424 footballers. Although the final sample (i.e., representing 84.8% response rate) was not up to 500 cases, it was relatively close to the recommended cases which guaranteed representativeness. The sampled players aged between 16 and 31 years (*M* = 22.36; *SD* = 3.53) and with 1-15 years of experience (*M* = 2.69; *SD* = 1.82). Nine of the participants representing 2.12% had obtained diploma and bachelor’s degrees, 33.72% (*n* = 143) had completed secondary school while 272 (64.16%) of the participants had obtained junior and primary level education. There were no strict criteria to qualify a player to play at the premier level. The basic qualification requirement was for the player to have a good record regarding discipline and performance.

Items used in this study were not translated into other local dialects because of three reasons; (1) many Ghanaian local languages have inconsistent forms and are not well written. Within a specific ethnic group, the same language can be written and spoken in different ways. The Fante language, for instance, has different forms, depending on the community one belongs to within the Ghanaian setting (Bronteng et al., 2020), (2) several researchers (see Owu-Ewie and Edu-Buandoh, 2014; Ackon, 2015; Bronteng et al., 2020; Dew Research, 2020) have confirmed that it is difficult for many Ghanaian youngsters to read, comprehend written information and/or write in their local languages. For example, Dew Research revealed that about 80% of the youth in Ghana are unable to read and write in their local languages, (3) prior informal information received from the participants during a familiarization visit showed that, although they could fluently speak their local languages, many of them could not adequately read and comprehend written information or write in their local languages. Based on these reasons, research assistants with a background in interpretations were recruited and trained to administer the instrument to the participants.

### Measures

#### The Stress Appraisal Measure

The 28-item Stress Appraisal Measure was used to assess six (6) appraisal (Peacock and Wong, 1990) mechanisms under primary and secondary cognitive appraisals. The specific appraisal mechanisms include; challenge (e.g., *To what extent can I become a stronger person because of this problem*?), threat (e.g., *Is this going to have a negative impact on me*?) and centrality (e.g., *Does this situation have important consequences for me*?). Higher-order dimensions such as controllable-by-others (e.g., *Is there anyone who can help me to manage this problem*?), controllable-by-self (e.g., *Do I have the ability to do well in this situation*?), and uncontrollable-by-anyone (e.g., *Is this a totally hopeless situation*?) are assessed for secondary appraisals. After measuring the relational meanings of primary and secondary appraisals, the general perceived stress that individuals reported were calculated. Items on the SAM are rated on a 5-point Likert type scale ranging from 1 = “*Not at all*,” 2 = “*Slightly*,” 3 = “*Moderately*,” 4 = “*Considerably*” to 5 = “*Extremely*.” Previous studies have reported sufficient Cronbach’s alpha coefficient values for the SAM ranging from 0.74 to 0.90 (Peacock and Wong, 1990; Gan and Anshel, 2006; Gan et al., 2009). The current study recorded Cronbach’s alpha coefficient values for primary appraisals to be 0.76 and 0.85 for secondary appraisals, respectively.

### Quality Control Strategy

#### Recruitment and Training of Research Assistants

The recruitment and training of research assistants for this research were largely guided by the quality control strategies adopted by Srem-Sai et al. (2021) in their study. Five research assistants were recruited as interpreters and/or translators taking the diverse languages which were spoken by the participants into consideration. Two of the research assistants were teaching assistants with a background in languages and translation who were employed in one of the Ghanaian public universities. The other three were postgraduate students pursuing programs in local languages. It must be emphasized that these research assistants had experience in instrument administration and data collection with years of experience ranging between 3 and 8 years. The research assistants were recruited strategically such that each assistant was fluent in at least two of the following languages: English, Dagbani, Ewe, Nzema, Fante, Ga, Bono, and Twi.

The assistants had two days of training on the administration of the survey instrument. First, the purpose together with the methodology of the study were discussed with the assistants. Copies of the SAM were made available to the assistants and the items were discussed one after the other. Particular attention was paid to the scale point indicating what they mean and what goes into each of the scale categories. This was done to ensure that all assistants understood the scale categories. The earlier discussions were done using the English language. After all the assistants were clear on the issues discussed, they were taken through how the items should be interpreted. Further, the assistants were also oriented on how to adhere to the required ethical considerations like volition, privacy, confidentiality, informed consent, and anonymity.

The training was climaxed with a two-stage pilot-testing which were carried out on the field. The purpose of the first pilot testing was to assess the degree of consistency among the research assistants in terms of interpreting the statements and the scale categories. To achieve this, the club coaches were contacted to purposefully select five players who were fluent in the Twi language. Each assistant administered the SAM using the Twi language to all the five purposefully sampled players. Using the Generalized Analysis of Variance (GENOVA) procedure, the data obtained from this stage were subjected to analysis to understand the extent of item-interpreter reliability (Brennan, 2011). The results yielded generalizability coefficients (*g*) of 0.84 and phi coefficient (Φ) of 0.81, indicating that the assistants showed a sufficient level of consistency among interpreters and across participants of the interpretations (Creswell, 2012). The second stage of the piloting sampled five GPL players who were purposefully selected based on their languages (i.e., Dagbani, Nzema, Bono, Ewe and Ga). The research assistants who were fluent in these Ghanaian languages administered the survey instrument to the five sampled players. This phase was observed and supervised by five supervisors who were lecturers teaching local language courses and also fluent in these languages. After each administration, the accuracy of interpretations was scored over 100 by the supervisors. A mean score of 82% was obtained which reflected sufficient accuracy in interpreting the items.

### Procedure

Reference number UCC/IRB/A/2016/794 was obtained after the Institutional Review Board of the University of Cape Coast gave ethical approval for the study to be conducted. The study participants were selected after a meeting was organized among the club Chief Executive Officers, managers, owners, coaches, and the footballers to discuss, familiarize and deliberate on the study’s rationale and significance. Adequate information was provided to all participants concerning their rights to anonymity, confidentiality of all responses given, and could withdraw from participating in the study at any time without any penalties. Participants were further informed and assured that the information they provide would be kept safely in the custody of only the researchers and was for academic purposes. Before collecting the data, each participant willingly endorsed a consent form, confirming their readiness to participate in the study. The study measure (SAM) was administered to all participants with the help of the research assistants. Answering the items on the instrument lasted between 15 and 20 min within 3 months for all clubs. About three-quarters, 74.5% (*n* = 316) of the participants expressed their inability to communicate effectively in the English Language so they were assisted during the data collection. Thus, assistance was given to such participants who could not read and comprehend the items in the English Language. This was done by interpreting the various items in their local dialects for easy response. Data were collected at the home camps of the various teams. Administered questionnaires were collected and sealed in envelopes for safekeeping.

### Statistical Analyses

The graded response model of the ordered polytomous item response theory family was used for the validation study (Samejima, 1969, 1997). The study focused on between-item multidimensional structure (Ye et al., 2019; Liang et al., 2021). The IRT PRO software (version 4.2) was used for the analysis (Cai et al., 2011b). The assumption of unidimensionality was relaxed due to the theoretical support of the multidimensionality of the SAM (Folkman and Lazarus, 1985, 1988; Peacock and Wong, 1990; Durak and Senol-Durak, 2013). The data were analyzed to understand the quality of the items on the SAM in Ghana by assessing the discrimination (slope) and difficulty parameters of the items, the amount of information each item adds to the construct, and the reliability of the instrument. Whereas the discrimination parameter provides an idea of how an item on a multi-trait scale is associated with the construct being measured; the difficulty parameter describes the threshold values at which a respondent will have a 50:50 chance of endorsing a particular category (Depaoli et al., 2018). Discrimination (slope) parameters greater than 0.50 depicts a good discrimination ability of the item (Baker, 2001). The response categories function appropriately in cases where the difficulty thresholds increase monotonically (Toland, 2014). Item level fit, which was assessed using the generalized S-X^2^ statistics (Kang and Chen, 2008) denotes whether the item measures any aspect of the construct of interest; when an item misfit, the possible causes of the misfit should be examined (e.g., item content, ambiguity, among others) (Depaoli et al., 2018). To assess whether the model fits the item, the item should have a p-value less than 1% (Stone and Zhang, 2003). The item information function was also examined to evaluate whether some of the items were redundant. Items with similar item information functions meant that they offered similar information to the latent trait (Toland, 2014).

The use of the multidimensional graded response model required that the data collected using the SAM meet certain assumptions. The SAM is a multidimensional instrument and was, thus, treated as such in statistical terms. This assumption was based on theoretical and empirical support that stress appraisal has multiple latent traits (Folkman and Lazarus, 1985, 1988; Peacock and Wong, 1990; Durak and Senol-Durak, 2013). The local dependency assumption was also tested to ensure that responses on each item is as a result of the construct being measured and not any other variable such as other items on the SAM, item wording, language barrier, interpreter effect, among others (Edelen and Reeve, 2007). The inspection of the local dependency matrix table (see Supplementary Appendix) showed that the local dependency statistics for 28 out 378 different pairs of items showed moderate (*n* = 25) to high (*n* = 3) level of dependency (Reeve et al., 2007). Items pairs with suspected local dependency issues include 2&12, 10&9, 11&12, 17&15, 27&28, 25&22, 28&23. Further investigation revealed sparseness in the local dependency results indicating that it was difficult pinpointing the specific item(s) with the issue. This coupled with the low proportion of local dependency (7.4%), it was assumed that the issue of dependency was not a major problem but only a concern for further careful investigation of other parameters (Cai et al., 2011a).

Further, a brief data description was required to warrant the use of the graded response model. Specifically, the number of observations falling into each of the ordered response category for each item was checked to ensure that they were adequate (Toland, 2014). This is necessary because adequate responses in each category per item improve the accuracy and precision of item parameters, and also help evaluate the extent of use of the various categories (De Ayala, 2009). Because of the validation of an existing instrument, there was the need to satisfy this assumption so that any shortfalls revealed after the validation could not be attributed to low responses on some response category. The descriptive analysis for the responses revealed that all the response categories for every item showed some level of adequacy of responses (see Table 1). Except for the “extremely” category for SAM 11 (“*Will the outcome of this situation be negative*?”) and SAM 27 (“*Does this situation have long-term consequences for me*?”) which had 9.7% and 9.4% of the responses, respectively, the rest had over 10% of the cases falling within the response categories. This notwithstanding, the cases were deemed sufficient (Toland, 2014).

**TABLE 1:** Frequency and percentage for category response per item.

Label	Statement	Not at all	Slightly	Moderately	Considerably	Extremely
SAM1	Is this a totally hopeless situation?	203 (47.9)	89 (21.0)	68 (16.0)	37 (8.7)	27 (6.4)
SAM2	Does this situation create tension in me?	128 (30.2)	126 (29.7)	79 (18.6)	56 (13.2)	35 (8.3)
SAM3	Is the outcome of this situation uncontrollable by anyone?	141 (33.3)	111 (26.2)	86 (20.3)	40 (9.4)	46 (10.8)
SAM4	Is there someone or some agency I can turn to for help if I need it?	64 (15.1)	113 (26.7)	95 (22.4)	77 (18.2)	75 (17.7)
SAM5	Does this situation make me feel anxious?	101 (23.8)	125 (29.5)	94 (22.2)	55 (13.0)	49 (11.5)
SAM6	Does this situation have important consequences for me?	86 (20.3)	98 (23.1)	95 (22.4)	78 (18.4)	67 (15.8)
SAM7	Is this going to have a positive impact on me?	79 (18.6)	82 (19.3)	89 (21.0)	101 (23.8)	73 (17.2)
SAM8	How eager am I to tackle this problem?	46 (10.8)	80 (18.8)	106 (25.0)	98 (23.1)	94 (22.2)
SAM9	How much will I be affected by the outcome of this situation?	58 (13.7)	119 (28.1)	108 (25.0)	90 (21.2)	51 (12.0)
SAM10	To what extent can I become a stronger person because of this problem?	73 (17.2)	93 (21.9)	100 (23.6)	99 (23.3)	59 (13.9)
SAM11	Will the outcome of this situation be negative?	118 (27.8)	105 (24.8)	88 (20.8)	72 (17.0)	41 (9.7)
SAM12	Do I have the ability to do well in this situation?	45 (10.6)	88 (20.8)	115 (27.1)	86 (20.3)	90 (21.2)
SAM13	Does this situation have serious implications for me?	77 (18.2)	124 (29.2)	110 (25.0)	66 (15.6)	47 (11.1)
SAM14	Do I have what it takes to do well in this situation?	53 (12.5)	75 (17.7)	114 (26.9)	103 (24.3)	79 (18.6)
SAM15	Is there help available to me for dealing with this problem?	83 (19.6)	95 (22.4)	113 (26.7)	66 (15.6)	67 (15.8)
SAM16	Does this situation tax or exceed my coping resources?	101 (23.8)	121 (28.5)	93 (21.9)	60 (14.2)	49 (11.6)
SAM17	Are there sufficient resources available to help me in dealing with this situation?	91 (21.5)	91 (21.5)	109 (25.7)	68 (16.0)	66 (15.3)
SAM18	Is it beyond anyone’s power to do anything about this situation?	129 (30.4)	87 (20.5)	96 (22.6)	63 (14.9)	49 (11.6
SAM19	To what extent am I excited thinking about the outcome of this situation?	75 (17.7)	93 (21.9)	122 (28.8)	86 (20.3)	48 (11.3)
SAM20	How threatening is this situation?	80 (18.8)	79 (18.6)	100 (23.6)	95 (22.4)	69 (16.3)
SAM21	Is the problem unresolvable by anyone?	134 (31.6)	85 (20.0)	78 (18.4)	73 (17.2)	54 (12.7)
SAM22	Wil I be able to overcome the problem?	55 (13.0)	75 (17.7)	95 (22.4)	101 (23.4)	98 (23.1)
SAM23	Is there anyone who can help me to manage this problem?	58 (13.7)	100 (23.6)	109 (25.7)	83 (19.6)	74 (17.5)
SAM24	To what extent do I perceive this situation as stressful?	61 (14.4)	92 (21.7)	121 (28.5)	68 (16.0)	82 (19.3)
SAM25	Do I have the skills necessary to achieve a successful outcome to this situation?	63 (14.9)	87 (20.5)	102 (24.1)	76 (17.9)	96 (22.6)
SAM26	To what extent does this event require coping efforts on my part?	74 (17.5)	105 (24.8)	123 (29.0)	66 (15.6)	58 (13.2)
SAM27	Does this situation have long-term consequences for me?	110 (25.9)	111 (26.2)	85 (20.0)	78 (18.4)	40 (9.4)
SAM28	Is this going to have a negative impact on me?	124 (29.2)	101 (23.8)	96 (22.6)	66 (15.6)	37 (8.7)

Different model fit indices are reported. The loglikelihood fit statistics showed a value of 17638.24. The reduced *M2* statistics for the multidimensional model was nonsignificant, *M2* = 17.32, *p = 0*.083. The RMSEA estimate was 0.032. The model fit indices supported the appropriateness of the model.

## Results

### Item Parameter Estimates

The item parameter estimates comprised two key features about the items: (1) the ability of the items to distinguish between respondents who possess a high level of the trait from those with a low level of the trait (item slope) (Toland, 2014); and (2) the level at which a participant with a particular latent trait has an equal chance of endorsing an item (e.g., *considerably* vs. *extremely*). The details of the results are shown in Table 2.

**TABLE 2:** Graded model item parameter estimates, logit: *a*(θ – *b*).

Label	*a*_1_ *(s.e)*	*a*_2_ *(s.e)*	*a*_3_ *(s.e)*	*a*_4_ *(s.e)*	*a*_5_ *(s.e)*	*a*_6_ *(s.e)*	*b*_1_	*s.e._*b*1_*	*b*_2_	*s.e._*b*2_*	*b*_3_	*s.e._*b*3_*	*b*_4_	*s.e._*b*4_*
**Factor 1: Threat (7-items)**														
SAM2	0.93 (0.13)	–	–	–	–	–	–1.02	0.18	0.52	0.12	1.63	0.22	2.94	0.39
SAM5	1.06 (0.14)	–	–	–	–	–	–1.29	0.18	0.19	0.10	1.31	0.17	2.30	0.28
SAM11	1.11 (0.14)	–	–	–	–	–	–1.05	0.16	0.11	0.10	1.10	0.15	2.38	0.28
SAM16	0.59 (0.11)	–	–	–	–	–	–10.66	2.51	–2.12	0.40	0.18	0.17	1.93	0.37
SAM20	0.92 (0.12)	–	–	–	–	–	–7.19	1.38	–1.85	0.25	–0.63	0.14	0.60	0.13
SAM24	0.79 (0.12)	–	–	–	–	–	–2.52	0.37	–0.81	0.17	0.87	0.17	2.03	0.30
SAM28	1.06 (0.14)	–	–	–	–	–	–0.99	0.16	0.15	0.10	1.28	0.17	2.59	0.32
**Factor 2: Challenge (4-items)**														
SAM7	–	0.90 (0.12)	–	–	–	–	–1.86	0.26	–0.61	0.14	0.48	0.13	1.99	0.27
SAM8	–	0.78 (0.12)	–	–	–	–	–2.98	0.44	–1.18	0.21	0.31	0.14	1.80	0.28
SAM10	–	0.60 (0.11)	–	–	–	–	–2.83	0.51	–0.79	0.22	0.96	0.23	3.26	0.58
SAM19	–	0.55 (0.11)	–	–	–	–	–11.26	2.74	–2.95	0.57	–0.81	0.23	1.46	0.31
**Factor 3: Centrality (4-items)**														
SAM6	–	–	1.13 (0.14)	–	–	–	–1.45	0.19	–0.25	0.10	0.76	0.12	1.81	0.21
SAM9	–	–	0.92 (0.12)	–	–	–	–2.29	0.30	–0.40	0.13	0.88	0.15	2.44	0.31
SAM13	–	–	1.18 (0.14)	–	–	–	–1.56	0.19	–0.08	0.10	1.07	0.14	2.13	0.24
SAM27	–	–	1.04 (0.13)	–	–	–	–1.23	0.18	0.09	0.10	1.11	0.16	2.54	0.31
**Factor 4: Controllable-by-self (5-items)**														
SAM12	–	–	–	0.75 (0.12)	–	–	–3.12	0.48	–1.12	0.21	0.56	0.15	1.97	0.31
SAM14	–	–	–	0.66 (0.11)	–	–	–3.15	0.53	–1.32	0.26	0.52	0.17	2.41	0.41
SAM22	–	–	–	0.61 (0.11)	–	–	–3.33	0.60	–1.40	0.29	0.26	0.17	2.15	0.40
SAM25	–	–	–	0.55 (0.11)	–	–	–3.35	0.65	–1.14	0.28	0.76	0.22	2.37	0.47
SAM26	–	–	–	0.85 (0.12)	–	–	–2.05	0.29	–0.38	0.13	1.26	0.20	2.52	0.35
**Factor 5: Controllable-by-others (4-items)**														
SAM4	–	–	–	–	0.62 (0.11)	–	–3.00	0.53	–0.55	0.19	1.06	0.23	2.70	0.47
SAM15	–	–	–	–	0.47 (0.10)	–	–3.13	0.71	–0.72	0.26	1.72	0.42	3.69	0.82
SAM17	–	–	–	–	0.32 (0.10)	–	–4.14	1.32	–0.88	0.41	2.55	0.83	5.51	1.73
SAM23	–	–	–	–	0.55 (0.11)	–	–3.58	0.69	–1.02	0.26	1.04	0.26	3.00	0.58
**Factor 6: Uncontrollable-by-anyone (4-items)**														
SAM1	–	–	–	–	–	0.70 (0.12)	–0.14	0.15	1.23	0.24	2.68	0.46	4.14	0.71
SAM3	–	–	–	–	–	0.72 (0.12)	–1.10	0.22	0.56	0.16	2.07	0.33	3.17	0.50
SAM18	–	–	–	–	–	0.62 (0.11)	–1.42	0.29	0.10	0.16	1.82	0.34	3.56	0.63
SAM21	–	–	–	–	–	0.54 (0.11)	–1.50	0.34	0.14	0.18	1.67	0.35	3.72	0.73

*a_1_- Threat dimension, a_2_- Challenge dimension, a_3_- Centrality, a_4_- Controllable-by-self dimension, a_5_- Controllable-by-others, a_6_- Uncontrollable-by-anyone.*

The results revealed that the majority of the items had good discrimination indices (slope parameter greater than 0.50). Item 5 (“*Does this situation make me feel anxious*?”), for example, had a slope parameter of 1.06 with a standard error of 0.14. Item 11 (“*Will the outcome of this situation be negative*?”) had an index of 1.11 and a standard error of −1.05. Two of the items (SAM 15, “*Is there help available to me for dealing with this problem*?”; SAM 17, “*Are there sufficient resources available to help me in dealing with this situation*?”) had low discrimination indices of 0.47 (SAM 15) and 0.32 (SAM 17) respectively. This suggests that these two items were poor in terms of distinguishing respondents with high latent traits and those with low latent traits. These items were captured under the *uncontrollable-by-anyone* dimension.

The difficulty parameter estimates revealed that generally the respondents who were low on the construct were more likely to endorse the “*not at all*” category whereas those who were high on the latent trait had higher chances of endorsing the “*extremely*” response option. Item 1 (SAM 2, “*Does this situation create tension in me?*”), for example, had difficulty thresholds of −1.02, 0.52, 1.63, and 2.94 for *b*_1_, *b*_2_, *b*_3_, and *b*_4_, respectively (see Table 2), indicating that respondents with a low latent trait are more likely to endorse the ‘*not at all*’ category compared to the “*slightly*,” “*comparably*,” and “*extremely”*. Item 5 (SAM 5, “*Does this situation make me feel anxious*?”) also had difficulty thresholds of −1.29, 0.19, 1.31, and 2.30 for *b*_1_, *b*_2_, *b*_3_, and *b*_4_, respectively. Generally, the difficulty threshold increased monotonically.

### Item Level Fit

The study examined the absolute fit of the model to each item by examining the level of equivalence between the predicted model and observed response frequencies based on item response category (Orlando and Thissen, 2000, 2003). Specifically, the study assessed the extent to which each item measure or belong to the construct being measured. Table 3 highlights the details of the results.

**TABLE 3:** S-*X*^2^ item level diagnostic statistics.

Dimensions	Label	*X* ^2^	*df*	Probability
Threat	SAM2	158.46	124	0.0199
	SAM5	132.26	125	0.3107
	SAM11	151.81	124	0.0454
	SAM16	198.51	134	0.0002*
	SAM20	171.88	133	0.0130
	SAM24	169.05	132	0.0163
	SAM28	169.56	114	0.0006*
Challenge	SAM7	140.38	135	0.3577
	SAM8	149.66	138	0.2347
	SAM10	166.86	142	0.0754
	SAM19	170.54	141	0.0456
Centrality	SAM6	141.79	130	0.2261
	SAM9	132.13	130	0.4309
	SAM13	135.48	127	0.2867
	SAM27	178.48	125	0.0012*
Controllable-by-self	SAM12	199.64	124	0.0001*
	SAM14	197.74	141	0.0012*
	SAM22	166.75	143	0.0849
	SAM25	159.74	133	0.0568
	SAM26	143.71	126	0.1336
Controllable-by-others	SAM4	181.43	140	0.0105
	SAM15	196.21	141	0.0015*
	SAM17	180.93	146	0.0002*
	SAM23	187.80	140	0.0044*
Uncontrollable-by-anyone	SAM1	165.48	107	0.0262
	SAM3	154.66	121	0.0210
	SAM18	169.21	129	0.0101
	SAM21	178.12	137	0.0104

*Significant at p < 0.01.

The outcome of the calibration results showed that the 28-item (SAM instrument) model generally had a satisfactory fit. This was because about 20 items had a non-significant probability value (Stone and Zhang, 2003). Eight items did not show adequate representatives by the estimated item parameter (see Table 3). These items were SAM 12 (“*Do I have the ability to do well in this situation?*”, *p = 0*.0001), SAM 14 (“*Do I have what it takes to do well in this situation*?,” *p* = 0.0001), SAM 15 (“*Is there help available to me for dealing with this problem*?”), SAM 16 (“*Does this situation tax or exceed my coping resources*?”), SAM 17 (“*Are there sufficient resources available to help me in dealing with this situation*?”), SAM 23 (“*Is there anyone who can help me to manage this problem*?”), SAM 27 (“*Does this situation have long-term consequences for me*?”), and SAM 28 (“*Is this going to have a negative impact on me*?”).

### Amount of Empirical Information Individual Item Contributes to the Latent Trait

The study examined the amount of information each item was contributing to the SAM scale and the location where such information can be located on the continuum. Items with less information need item content inspection, modification or removal. Also, the item information function distribution provides knowledge about the redundant items. Table 4 highlights the details of the result.

**TABLE 4:** Item information function values at 15 values of θ from −2.8 to 2.8.

Item	Label	−2.8	−2.4	−2.0	−1.6	−1.2	−0.8	−0.4	−0.0	0.4	0.8	1.2	1.6	2.0	2.4	2.8
**Threat**																
2	SAM2	0.12	0.15	0.18	0.21	0.24	0.25	0.26	0.27	0.27	0.28	0.28	0.28	0.27	0.26	0.25
5	SAM5	0.16	0.20	0.25	0.29	0.32	0.33	0.34	0.35	0.35	0.35	0.35	0.35	0.33	0.31	0.28
11	SAM11	0.14	0.18	0.24	0.29	0.34	0.36	0.38	0.39	0.39	0.39	0.38	0.38	0.37	0.34	0.31
16	SAM16	0.09	0.09	0.10	0.10	0.10	0.11	0.11	0.11	0.11	0.11	0.11	0.11	0.11	0.11	0.11
20	SAM20	0.19	0.21	0.23	0.25	0.26	0.26	0.27	0.27	0.27	0.26	0.26	0.25	0.24	0.22	0.19
24	SAM24	0.17	0.18	0.19	0.19	0.19	0.20	0.20	0.20	0.20	0.20	0.19	0.19	0.18	0.17	0.15
28	SAM28	0.13	0.17	0.22	0.27	0.31	0.33	0.35	0.35	0.36	0.36	0.36	0.35	0.34	0.33	0.30
**Challenge**																
7	SAM7	0.18	0.20	0.23	0.24	0.25	0.26	0.26	0.26	0.26	0.25	0.25	0.24	0.23	0.21	0.18
8	SAM8	0.17	0.18	0.19	0.19	0.19	0.19	0.19	0.19	0.19	0.19	0.18	0.18	0.17	0.15	0.13
10	SAM10	0.10	0.11	0.11	0.11	0.11	0.11	0.11	0.11	0.11	0.11	0.11	0.11	0.11	0.11	0.10
19	SAM19	0.09	0.09	0.09	0.10	0.10	0.10	0.10	0.10	0.10	0.10	0.10	0.10	0.10	0.09	0.09
**Centrality**																
6	SAM6	0.19	0.25	0.30	0.35	0.38	0.39	0.40	0.40	0.41	0.40	0.40	0.38	0.35	0.30	0.24
9	SAM9	0.21	0.23	0.25	0.25	0.26	0.26	0.26	0.27	0.27	0.27	0.27	0.26	0.25	0.24	0.22
13	SAM13	0.21	0.28	0.34	0.38	0.40	0.42	0.42	0.43	0.43	0.43	0.43	0.42	0.40	0.36	0.31
27	SAM27	0.15	0.19	0.24	0.28	0.30	0.32	0.33	0.34	0.34	0.34	0.34	0.33	0.32	0.31	0.28
**Controllable-by-self**																
12	SAM12	0.16	0.17	0.17	0.17	0.17	0.18	0.18	0.18	0.18	0.18	0.17	0.17	0.16	0.15	0.13
14	SAM14	0.13	0.13	0.14	0.14	0.14	0.14	0.14	0.14	0.14	0.14	0.14	0.13	0.13	0.12	0.12
22	SAM22	0.11	0.11	0.11	0.12	0.12	0.12	0.12	0.12	0.12	0.11	0.11	0.11	0.11	0.10	0.09
25	SAM25	0.09	0.09	0.09	0.10	0.10	0.10	0.10	0.10	0.10	0.10	0.09	0.09	0.09	0.09	0.08
26	SAM26	0.17	0.19	0.20	0.21	0.22	0.22	0.22	0.22	0.23	0.23	0.22	0.22	0.22	0.21	0.19
**Controllable-by-others**																
4	SAM4	0.11	0.11	0.12	0.12	0.12	0.12	0.12	0.12	0.12	0.12	0.12	0.12	0.12	0.11	0.11
15	SAM15	0.06	0.07	0.07	0.07	0.07	0.07	0.07	0.07	0.07	0.07	0.07	0.07	0.07	0.07	0.07
17	SAM17	0.03	0.03	0.03	0.03	0.03	0.03	0.03	0.03	0.03	0.03	0.03	0.03	0.03	0.03	0.03
23	SAM23	0.09	0.09	0.09	0.09	0.09	0.09	0.09	0.09	0.09	0.09	0.09	0.09	0.09	0.09	0.09
**Uncontrollable-by-anyone**																
1	SAM1	0.06	0.07	0.08	0.10	0.11	0.12	0.13	0.14	0.15	0.15	0.15	0.15	0.15	0.15	0.15
3	SAM3	0.09	0.11	0.12	0.13	0.14	0.15	0.16	0.16	0.16	0.16	0.16	0.16	0.16	0.16	0.15
18	SAM18	0.08	0.09	0.10	0.11	0.11	0.12	0.12	0.12	0.12	0.12	0.12	0.12	0.12	0.12	0.12
21	SAM21	0.07	0.07	0.08	0.08	0.09	0.09	0.09	0.09	0.09	0.09	0.09	0.09	0.09	0.09	0.09
Test Information:		4.54	5.07	5.56	5.97	6.26	6.44	6.55	6.61	6.64	6.64	6.59	6.50	6.32	6.02	5.60
Expected s.e.:		0.47	0.44	0.42	0.41	0.40	0.39	0.39	0.39	0.39	0.39	0.39	0.39	0.40	0.41	0.42

*Marginal reliability for response pattern scores: 0.85; −2loglikelihood: 35700.05, p > 0.001.*

**FIGURE 2:**
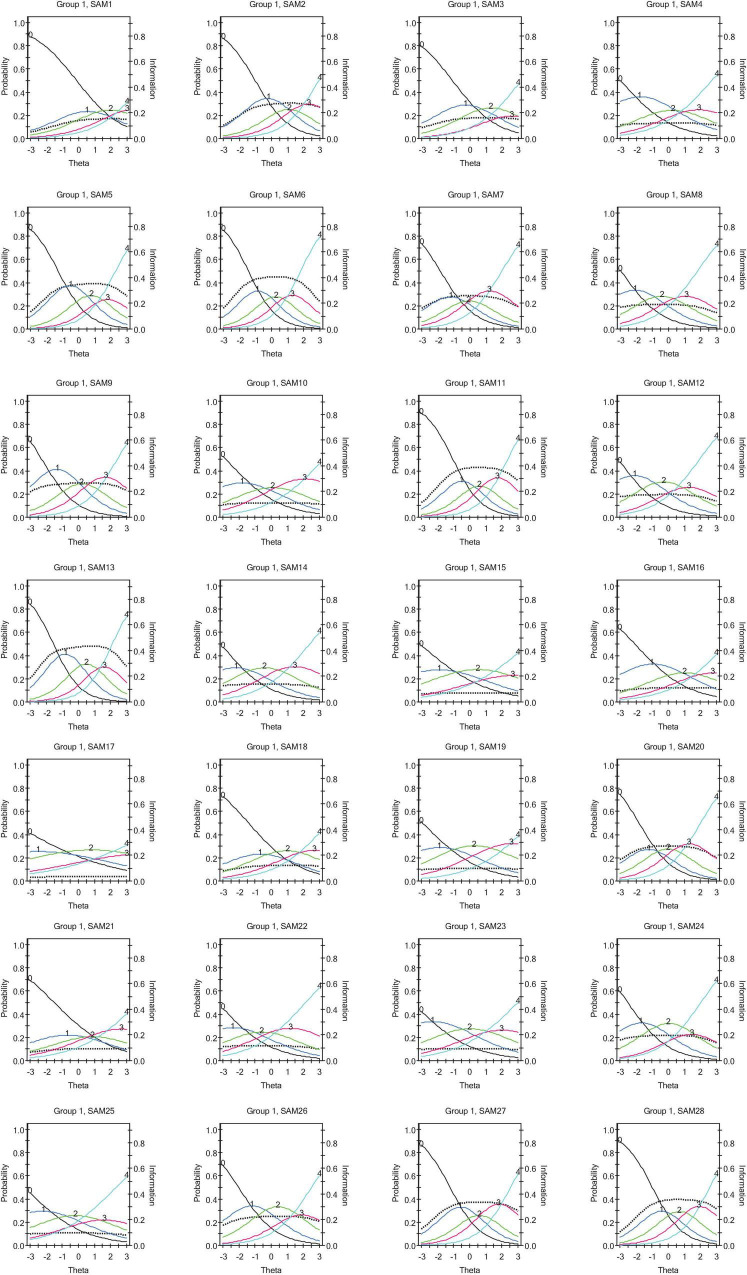
Item characteristics and information curve.

The analysis showed that 4 items contributed little empirical information to the measurement of stress appraisal of the participants. SAM 17 (“*Are there sufficient resources available to help me in dealing with this situation?*”) had the least information contribution, followed by SAM 15 (“*Is there help available to me for dealing with this problem?*”), SAM 21 (“*Is the problem unresolvable by anyone?*”), and finally SAM 23 (“*Is there anyone who can help me to manage this problem?*”) (see Table 4). SAM 17, for example, had a stable item information function value of 0.03 at 15 values of the latent trait from −2.8 to 2.8. SAM 15 also had information function values from 0.06 t0 0.07 at 15 values of the latent trait from −2.8 to 2.8. For SAM 21, item information function estimates ranged from 0.07 to 0.09, and the information function value of 0.09 was consistent across SAM 23 at 15 values of the latent trait from −2.8 to 2.8 (see Table 4, also see item trace graph, Figure 2).

Further, other pairs of items were found to provide similar information to the measurement of the construct. If two items offer similar information to latent trait, then one of the items is considered redundant (i.e., do not add anything new to the measure). For example, SAM 10 (“*To what extent can I become a stronger person because of this problem?*”) and SAM 16 (“*Does this situation have important consequences for me?*”) were found to offer nearly identical information in the measurement of stress appraisal. Other items which had similar information functions were SAM 21 (“*Is the problem unresolvable by anyone?*”) and SAM 23 (“*Is there anyone who can help me to manage this problem?*”), and SAM 25 (“*Do I have the skills necessary to achieve a successful outcome to this situation?*”) and SAM 19 (“*To what extent am I excited thinking about the outcome of this situation?*”).

Inspecting the item characteristic curves for the items, it was observed that the 5-point Likert scale appeared problematic. Taking SAM 1, for example, the option 1 (“*slightly*”) did not show much efficiency in discriminating between different abilities as compared to option 2 (“*moderately*”). Other items like SAM 4, SAM 15, SAM17, and SAM 21 had problems with the scale options 1 and 3.

**FIGURE 3:**
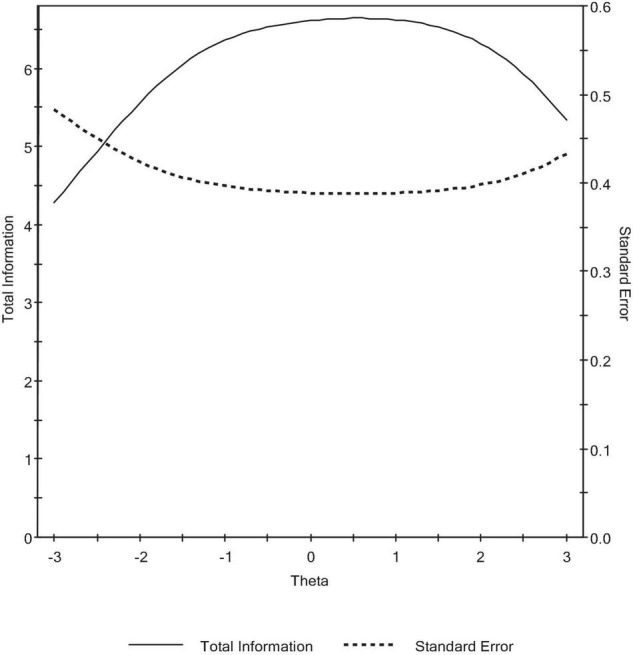
Total information function.

**FIGURE 4:**
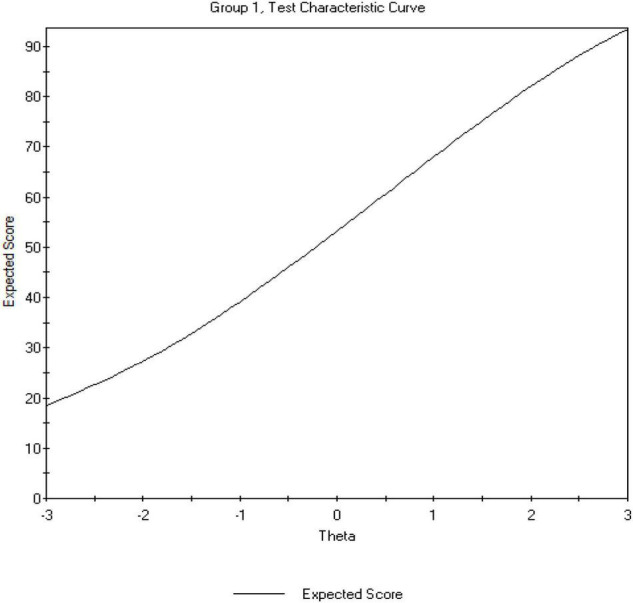
Test characteristic curve.

The total information function, which is the function of the specific item quality and the number of items, was also examined. As can be observed in Figure 3, the test information function increased monotonically with decreasing standard error. This yielded a reliability estimate of 0.85, which supports that there is some level of precision for the entire region covered by the items (Sireci et al., 1991; Kim and Feldt, 2010). This level of precision was also supported by the test characteristic curve, which reflects the relationship between ability and true score. Increasing ability level results in increasing true score. This suggests a high level of consistency between predicted ability and observed ability. For example, an ability value of −1 corresponds to an expected score of ≈40, and an ability level of 1 reflects a true score of ≈65 (see Figure 4).

## Discussion

This study sought to validate the stress appraisal measure among male football players in the Ghana Premier League using the multidimensional item response theory. Results revealed that each of the items on the SAM scale showed evidence of a nonlinear relationship with the latent trait (stress appraisal), even though two of the items do not meet the recommended 0.50 slope index. For these two items, one of them had a coefficient of 0.47. Generally, the results imply that the items were able to discriminate among the respondents in terms of their stress levels. Thus, the items could differentiate between respondents who reported high levels of stress as against those who indicated low stress levels. The ultimate goal in any measurement situation be it stress, depression, achievement, among others, is to be able to differentiate among those who are high on the trait and those who are low. The items on the SAM validated in the current study reflected good proxies for the measurement of stress among professional footballers in Ghana.

The results further revealed that the majority of the items fit the data based on the S-*X*^2^ Item-level statistics, whereas a few others appeared not to be a good fit based on the *p-*values. This was not so much of a problem knowing the estimation of the *p*-values is influenced by the sample sizes. Additionally, the complementary model-data fit as indicated by the −2loglikelihood suggested a good fit. Few of the items appeared to be redundant, but in all, the SAM was somewhat reliable. For example, an item under the *controllable-by-self* dimension and another measuring *challenge* domain provided similar information to the measurement of the construct. The SAM provided maximum information at ability groups of 0.4 and 0.8. The SAM showed that increased ability level results in an increased true score, and this suggests a high level of consistency between predicted and observed abilities.

The findings of the current validation showed that the response categories for the items on the SAM scale functioned fairly. Generally, it is expected that the probability of endorsing “*not at all*” category should be high among the respondents who are less on the latent trait, whereas the probability of endorsing “*extremely*” response category would be high on the trait. Notably, the 5-point Likert scale of the SAM seemed not to be appropriate with the sample used as there are traces of poor scale functioning for some items. That is, some of the scale options (i.e., options 1 and 3) appeared problematic for a number of the items. This suggests that the response format for the scale may be too few, limiting adequate differentiation or too many response options thereby overburdening respondents (Weng, 2004). From this premise, a well-functioning response scale of SAM is required to provide a good psychometric indicator in terms of its utility in evaluating stress appraisals across different samples (Lozano et al., 2008; Culpepper, 2013). This calls for a further investigation of the appropriateness of the response format for the SAM. Perhaps, different scale options will be appropriate for different samples, even when the same instrument is used (Naemi et al., 2009; Kutscher et al., 2017). To have a comprehensive view of the utility of the SAM in Ghana, future studies should adopt mixed item response model to examine scale usage appropriateness.

The evidence gathered from this study supports the applicability of the SAM to football players in the Ghanaian setting, although much calibration still needs to be done. Most especially, this validation study supported the six-factor structure of SAM originally found by Peacock and Wong (1990). According to previous validation studies (e.g., Roesch and Rowley, 2005; Durak and Senol-Durak, 2013), the 6-factor structure of SAM is only appropriate for the adult population and not for adolescents (Rowley et al., 2005) or students (Roesch and Rowley, 2005) sample because some of the dimensions, particularly the centrality sub-scale, require more complex processes to aid in the appraisal. For example, Rowley et al. (2005) argued that the centrality sub-scale is not appropriate for adolescents since a higher cognitive pattern of responses is required. Anshel et al. (1997), however, were of the view that changes in sample characteristics are key in determining the appropriate factor structure. The sample for this study can be considered as adult population because these soccer players in the GPL are mature enough with some aged around 30 years. Besides, the population was “non-elite” and thus, well-trained interpreters were recruited to administer the survey instrument. This could have potentially led to well-explained items and hence, respondents finding it easy to respond to the items which previous studies have identified as requiring complex cognitive operations. In contrast, the studies available (see Peacock and Wong, 1990; Roesch and Rowley, 2005; Rowley et al., 2005; Durak and Senol-Durak, 2013) used elite population and did not use interpreters; the respondents in these studies read and responded to the items on their own. It is not therefore surprising that the 6-factor structure was supported in this study due to the sample characteristics.

This notwithstanding, the controllable by others dimension had the majority of its items either redundant, having poor discrimination, providing very little information on the measurement of the construct, or appeared not to belong to the proxies of the construct being measured. This was inconsistent with what other previous studies have found. Peacock and Wong (1990), for instance, found that the items under the challenge and uncontrollable-by-anyone dimensions do not have strong covariances, indicating that some of the items used as proxies under these sub-scales were not contributing much to the measurement of the construct. Other scholars like Roesch and Rowley (2005) also confirmed the low internal consistency of items under the uncontrollable-by-anyone. Perhaps, these reported inconsistencies in the factor structure found in this study and previous studies could be attributed to the sample characteristics such as gender, age, educational level, occupation, among others (Anshel et al., 1997), cultural variables such that values and norms influence on construal of others, self, and the interplay between others and self (Markus and Kitayama, 1991), and statistical approach to the instrument validation. Previous validation of SAM adopted the weak measurement theory (CTT) procedures whereas this study employed a strong measurement theory (i.e., multidimensional graded response model) (Samejima, 1997; Embretson and Reise, 2000; Kamata and Bauer, 2008; Raykov and Marcoulides, 2011).

The findings of this research contribute significantly to the discourse on the adoption and utility of the SAM in the Ghanaian setting, particularly, using football players. By the outcome, scholars in sport psychology would be guided on the utilization of the SAM across different populations. The concept of stress appraisal and its measurement is not consistent across different populations and cultures (Markus and Kitayama, 1991).

## Limitations and Future Research Direction

The validation of any instrument in a particular context is not a single phased approach and thus, several pieces of calibrations or testing need to be carried out. Hence, the outcome of this study should not be taken as a full reflection of the validity of SAM in the Ghanaian context but should only act as a precursor to understand and guide the utility of the SAM. Therefore, further validation is required to establish the appropriateness of the SAM in Ghana and perhaps, other African countries with similar homogeneous population characteristics. Although researchers in Ghana in the field of stress are not discouraged from using the SAM questionnaire, the instrument should be re-validated before being used. Thus, the content of all the items should be inspected, paying attention to those items which were flagged as quite problematic.

Future validation studies in Ghana should include female participants to provide comprehensive information about the instrument. Item differential analysis should also be carried out in future studies. Further studies should assess whether some sub-scales of the SAM are state-like domains (can be changed by intervention) or trait-like subscales (cannot be changed) (Ye et al., 2020a). This aproach would inform the adoption/adaption of the SAM for intervention studies. Also, we suggest that the Minimum Clinical Important Difference of this instrument should be further estimated to facilitate its application in intervention studies that would adopt the SAM scale (Ye et al., 2020b).

## Conclusion

This research revealed promising applicability of the SAM questionnaire among male footballers who participated in the premier league in Ghana. Generally, the scale categories (5-point scale; *not at all*, *slightly*, *moderately*, *considerably*, and *extremely*) functioned fairly, with appreciable reliability estimates, and acceptable item parameters. This notwithstanding, there is the need for scholars to continuously validate the SAM in Ghana and with diverse populations to widen its generalization.

## Data Availability Statement

The raw data supporting the conclusions of this article will be made available by the authors, without undue reservation.

## Ethics Statement

The studies involving human participants were reviewed and approved by reference number UCC/IRB/A/2016/794 was obtained after the Institutional Review Board of the University of Cape Coast gave ethical approval for the study to be conducted. The patients/participants provided their written informed consent to participate in this study.

## Author Contributions

MS-S, FQ, and JH conceived the idea. FQ performed the analysis. MS-S, FQ, JH, FA, JF, PO, and TS prepared the initial draft of the manuscript. All authors thoroughly revised and approved the final version of the manuscript.

## Conflict of Interest

The authors declare that the research was conducted in the absence of any commercial or financial relationships that could be construed as a potential conflict of interest.

## Publisher’s Note

All claims expressed in this article are solely those of the authors and do not necessarily represent those of their affiliated organizations, or those of the publisher, the editors and the reviewers. Any product that may be evaluated in this article, or claim that may be made by its manufacturer, is not guaranteed or endorsed by the publisher.
