# Supplementary material for: Re-assessing the Psychometric Properties of Stress Appraisal Measure in Ghana Using Multidimensional Graded Response Model
Source: Front Psychol. 2022 May 19;13:856217. doi: 10.3389/fpsyg.2022.856217 (PMC9161214; doi:10.3389/fpsyg.2022.856217)
Supplement: Supplementary file 1 [file Data_Sheet_1.pdf]

SUPPLEMENTARY OUTPUT

APPENDIX A – LOCAL INDEPENDENCE

Label	X^2	1	2	3	4	5	6	7	8	9	10	11	12	13	14	15	16	17	18	19	20	21	22	23	24	25	26	27
**SAM1**	0.0																											
**SAM2**	0.1	2.0																										
**SAM3**	0.1	1.0	0.6																									
**SAM4**	0.1	1.6	3.1	2.7																								
**SAM5**	0.2	3.1	3.1	0.8	0.9																							
**SAM6**	0.2	4.2	2.4	-0.1	1.9	1.6																						
**SAM7**	0.1	0.3	2.0	2.4	4.9	1.9	4.4																					
**SAM8**	0.3	-0.8	0.3	3.0	2.2	0.8	0.2	1.5																				
**SAM9**	0.1	-0.6	0.3	0.9	1.0	1.6	4.6	-0.5	3.5																			
**SAM10**	0.0	0.8	1.0	2.2	4.9	4.6	3.3	0.8	2.2	8.6																		
**SAM11**	0.2	1.9	-0.7	0.5	3.3	0.1	2.9	3.0	4.7	2.8	5.8																	
**SAM12**	0.2	3.8	6.7	1.1	4.9	4.9	9.9	4.5	3.7	4.6	3.6	10.9																
**SAM13**	0.4	2.2	6.1	1.5	4.0	1.7	9.6	4.9	4.9	4.0	3.5	1.9	8.1															
**SAM14**	0.3	2.9	3.7	0.6	1.7	3.5	6.0	4.7	2.0	2.1	4.0	9.5	9.9	2.8														
**SAM15**	0.1	2.6	4.4	0.4	3.1	2.8	3.2	2.2	3.0	1.8	3.0	3.9	6.0	4.1	4.2													
**SAM16**	0.0	1.7	3.0	-1.8	-0.1	0.8	1.9	3.0	2.0	0.7	3.3	1.2	2.9	2.5	2.1	3.6												
**SAM17**	0.0	1.8	0.6	0.8	1.6	0.0	3.7	1.3	1.0	0.6	0.9	1.2	2.0	5.3	3.8	7.6	2.3											
**SAM18**	0.0	0.2	1.6	1.0	1.7	4.1	4.5	4.4	2.8	0.4	1.9	1.6	5.0	4.6	0.3	3.7	4.2	4.6										
**SAM19**	0.1	1.6	2.9	1.2	1.9	0.9	2.4	1.0	4.2	7.8	1.2	9.2	6.0	3.7	3.8	3.6	4.3	3.1	0.3									
**SAM20**	0.1	1.1	2.8	0.2	0.2	0.8	5.3	3.4	2.6	1.1	2.4	0.6	3.5	6.6	4.2	1.9	3.1	6.0	2.1	4.8								
**SAM21**	0.0	-0.2	1.2	1.6	3.4	2.6	2.9	1.7	2.4	0.5	3.0	2.8	1.9	3.2	2.4	2.8	1.3	2.5	6.9	0.3	1.4							
**SAM22**	0.1	1.6	2.1	-0.5	3.2	1.9	4.0	1.1	2.3	3.9	1.3	-0.2	4.9	3.1	6.1	2.1	4.4	3.4	4.9	3.7	0.1	3.9						
**SAM23**	0.0	4.5	2.7	2.2	4.9	2.5	4.7	-0.1	4.5	6.1	4.8	3.9	4.3	4.0	2.6	9.2	2.3	2.6	4.6	1.7	9.7	2.3	4.9					
**SAM24**	0.0	0.7	2.2	2.9	0.2	0.1	1.5	-0.8	3.9	0.2	1.3	4.7	1.5	3.6	3.1	3.8	3.5	1.1	1.3	3.4	2.4	1.9	0.8	0.7				
**SAM25**	0.0	-0.0	4.7	2.9	-0.5	4.9	4.9	2.9	-0.9	1.5	1.2	3.9	2.3	3.3	6.7	6.0	4.3	4.6	2.7	4.7	3.8	2.5	8.9	4.9	3.5			
**SAM26**	0.2	2.0	3.0	0.7	1.3	1.9	3.4	1.1	0.2	3.4	2.3	1.1	4.7	4.0	3.0	4.6	4.1	3.2	2.0	4.7	0.1	4.8	1.8	3.6	1.2	4.6		
**SAM27**	0.0	0.0	2.2	1.4	1.9	0.3	9.9	3.6	1.7	1.3	2.1	3.9	9.2	7.9	11.1	6.4	3.2	2.8	4.8	4.9	1.6	2.3	4.8	4.8	2.6	4.9	0.8	
**SAM28**	0.2	0.8	1.6	3.3	1.9	4.4	6.5	4.0	0.8	1.9	4.9	4.5	7.2	3.3	8.3	4.3	3.6	4.9	3.9	3.7	3.9	6.5	4.6	6.7	1.9	4.1	3.7	13.2
